# Chromosomal microarray testing yield in 829 cases of microcephaly: a clinical characteristics-based analysis for prenatal and postnatal cases

**DOI:** 10.1007/s00404-024-07388-3

**Published:** 2024-03-18

**Authors:** Rivka Sukenik-Halevy, Nir Mevorach, Lina Basel-Salmon, Reut Tomashov Matar, Sarit Kahana, Kochav Klein, Ifaat Agmon-Fishman, Michal Levy, Idit Maya

**Affiliations:** 1 Genetic Institute, Meir Medical Center, Kfar Saba, Israel; 2 School of Medicine, Faculty of Medical and Health Sciences, Tel Aviv University, Tel Aviv, Israel; 3 Recanati Genetic Institute, Rabin Medical Center, Petah Tikva, Israel; 4 Felsenstein Medical Research Center, Rabin Medical Center, Petah Tikva, Israel; 5 Pediatric Genetics Unit, Schneider Children’s Medical Center, Petah Tikva, Israel

**Keywords:** Microcephaly, Chromosomal microarray, Malformations

## Abstract

**Introduction:**

Microcephaly, characterized by abnormal head growth, can often serve as an initial indicator of congenital, genetic, or acquired disorders. In this study, we sought to evaluate the effectiveness of chromosomal microarray (CMA) testing in detecting abnormalities in both prenatal and postnatal cases of microcephaly.

**Materials and methods:**

CMA Testing: We conducted CMA testing on 87 prenatally-detected microcephaly cases and 742 postnatal cases at a single laboratory. We evaluated the CMA yield in relation to specific clinical characteristics.

**Results:**

In prenatal cases, pathogenic and likely pathogenic (LP) results were identified in 4.6% of cases, a significantly higher rate compared to low-risk pregnancies. The male-to-female ratio in this cohort was 3, and the CMA yield was not influenced by gender or other clinical parameters. For postnatal cases, the CMA yield was 15.0%, with a significantly higher detection rate associated with dysmorphism, hypotonia, epilepsy, congenital heart malformations (CHM), learning disabilities (LD), and a history of Fetal growth restriction (FGR). No specific recurrent copy number variations (CNVs) were observed, and the rate of variants of unknown significance was 3.9%.

**Conclusions:**

The yield of CMA testing in prenatal microcephaly is lower than in postnatal cases (4.6% vs. 15%). The presence of microcephaly, combined with dysmorphism, hypotonia, epilepsy, CHD, LD, and FGR, significantly increases the likelihood of an abnormal CMA result.

**Supplementary Information:**

The online version contains supplementary material available at 10.1007/s00404-024-07388-3.

## Introduction

Microcephaly is often considered within the broader spectrum of phenotypic features associated with underlying congenital, genetic, or acquired conditions. The diagnosis of prenatal microcephaly relies on the use of various sonographic growth charts specifically designed for evaluating fetal head biometry. However, it is important to note that there remains ongoing debate and controversy surrounding the precise definitions and criteria for classifying microcephaly [1, 2].

Prenatal microcephaly is considered a group I malformation of cortical development diagnosed according to ultrasonographic skull measurements [3]. Microcephaly present at birth is defined as primary microcephaly, while microcephaly that develops postnatally is defined as secondary microcephaly [4]. The definition of microcephaly is an occipitofrontal circumference (OFC) more than three standard deviations (SD) below the mean for a given age, gender, and gestation. However, some use a cutoff of more than two SD below the appropriate mean [4–6].

Microcephaly may have a constitutional relationship with ethnic background. The etiology of microcephaly is highly heterogeneous. Genetic syndromes are known to play a significant role in their etiology, as microcephaly is a component of the phenotype of numerous syndromes. This includes syndromes associated with copy number variation (CNV) detectable through chromosomal microarray analysis (CMA) [7–9] as well as monogenic syndromes [7–10].

In the last decade, CMA has replaced conventional karyotyping and become the primary test for prenatal diagnosis of fetal congenital anomalies detected by ultrasound [11].

Microcephaly is associated with several copy number variants detectable by CMA. Most of these cases are not isolated [12–15]. The accepted evaluation of microcephaly detected prenatally, frequently includes genetic counseling and recommendation for invasive prenatal testing by CMA. However, the exact frequency of clinically significant CMA findings in this scenario has not yet been reported. Thus, the objective of this study was to assess the contribution of CMA analysis in cases of abnormal head circumference (HC), in both isolated and non-isolated cases.

## Methods

### Data collection

The study’s inclusion criteria consisted of individuals diagnosed with microcephaly who had undergone CMA analysis. The study encompassed a total of 829 cases, including 87 prenatal cases and 742 postnatal cases. All these cases had undergone CMA testing at a single clinical laboratory between January 2014 and September 2020. Samples for testing were obtained from various medical centers and private facilities, all of which met the inclusion criteria for this study.

Comprehensive data, including the indication for testing, patient characteristics, patient and family history, ethnic background, and test results, were gathered from genetic counseling summary letters and lab requisition forms, which were obtained for all cases.

In the prenatal context, testing was indicated for cases in which fetal microcephaly was identified via ultrasound, and amniotic fluid samples were used for prenatal testing. Postnatal testing was conducted using peripheral blood samples.

All participants were offered prenatal screening in accordance with the guidelines provided by the Israeli Ministry of Health. Testing is covered by Health Maintenance Organizations (HMOs). Routine prenatal screening included nuchal translucency (NT) measurements between 11 and 13 weeks of gestation, followed by detailed early fetal anomaly scans at 14–16 weeks and late fetal anomaly scans at 20–24 weeks of gestation.

In addition, when deemed necessary, fetal echocardiography was performed by either experts in fetal echocardiography or pediatric cardiologists.

Well-documented approach to both anatomical and cardiac ultrasound assessments described in recent review papers [16, 17].

In addition, maternal biochemical screening tests (triple test) are routinely conducted during both the first and second trimesters, with all relevant data being meticulously recorded.

It is important to note that noninvasive prenatal testing (NIPT) is not included as a routine component of prenatal follow-up in Israel.

### Chromosomal microarray analysis (CMA)

CMA was performed using two platforms:

Tests performed during the years January 2013 through April 2017 were done using Human OmniExpress-24 v1.0 BeadChip (Illumina Inc., San Diego, CA, USA), which contains 716,503 genome-wide markers at an average spacing of 4 kb. It targets a minor allele frequency of 5%, as reported in the HapMap data. It includes SNPs within 10 kb of RefSeq genes, nonsynonymous SNPs (NCBI annotated), MHC/ADME SNPs, and sex chromosomes. DNA amplification, tagging and hybridization were performed according to the manufacturer’s protocol, aided by the Tecan Freedom Evo (Tecan, Mannedorf, Switzerland). The array slides were scanned on an iScan Reader (Illumina, Inc.). All data collected were evaluated using Illumina Genome Studio v2011.1 software and genome build GRCh37/hg19. Data were analyzed using Nexus Copy Number 7.5 (BioDiscovery, El Segundo, CA, USA).

The latter scans performed during May 2017 through September 2020 were performed using a CytoScan 750 K array (Affymetrix, Santa Clara, CA, USA). This platform is composed of 550,000 non-polymorphic copy number variant (CNV) probes and more than 200,000 single nucleotide polymorphism probes, with an average resolution of 100 Kb. Array data were analyzed using Chromosome Analysis Suite (ChAS) v2.1 Software (Affymetrix) (genome build GRCh37/hg19).

Microarray findings were reviewed at the time of data collection by one of the authors (I.M.) and categorized into four categories in accordance with the recommended guidelines from the American College of Medical Genetics [18]: Array results were considered normal when only benign and likely benign variants were detected., array results were considered positive or clinically significant finding when pathogenic or /likely pathogenic (LP) variants were detected., Variants of unknown clinical significance (VUS) and variants with low penetrance were included only if they should have been reported according to the guidelines determined by Israeli Society of Medical Geneticists; i.e., deletions of 1 Mb or larger and duplications of 2 Mb or larger. Microarray results were also categorized into “karyotype detectable” (i.e., copy number variants at least 10 MB in size) or not “karyotype-detectable” to assess the incremental yield of CMA over karyotype. Analysis of additional CNVs (second hits) was assessed in our cohort of cases with pathogenic/ LP results.

For the prenatal cohort we compared the CMA yield to the background risk in low-risk pregnancies in a cohort of 5541 CMA testes performed in pregnancies with no malformations detected by ultrasound performed in our lab [19]. The detection rate for this cohort was 1.4% (78 cases).

### Statistical analysis

Fisher’s Exact Test was used to test the differences between yield of CMA in relation to different parameters and compared to the background risk, with *P* < 0.05 considered statistically significant. Python statistics library version 3.5.1 (scipy.stats) was used for statistical analysis.

### Alignment with STROBE guidelines

We conducted this observational study with a commitment to transparent and comprehensive reporting, following the STROBE (STrengthening the Reporting of OBservational studies in Epidemiology) guidelines established by the Equator Network.

## Results

**Fig. 1:**
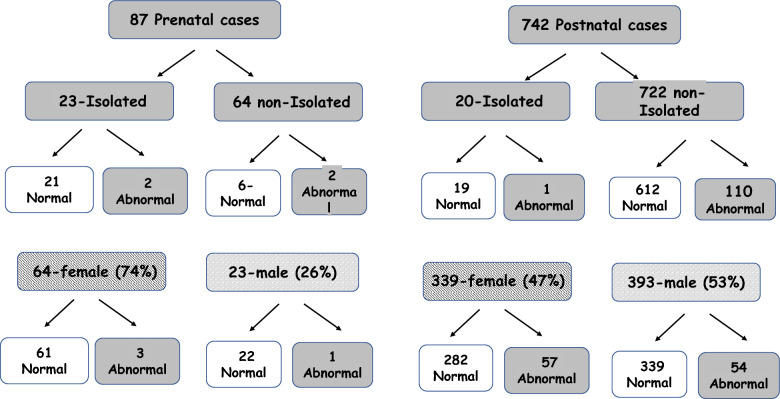
The yield of CMA testing in a cohort of 829 cases of microcephaly

Among the 829 CMA tests conducted in cases of microcephaly throughout the study period, whether identified prenatally or postnatally, 115 (13.9%) revealed pathogenic/ LP outcomes. The yield of CMA testing in our cohort is presented in Fig. 1. Among the 116 cases, 18% of these abnormalities were amenable to detection through karyotyping.

### CMA for prenatally-detected microcephaly.

Out of the 87 cases included in the prenatal cohort, 4 (4.6%) exhibited a pathogenic result, of which only one could potentially have been identified through karyotyping. Among these cases, two were isolated, each with a unique detected CNV, with one case also having a secondary CNV of low penetrance. In addition, one case with ventriculomegaly and severe early fetal growth restriction (FGR) was diagnosed with triploidy, while another case with microcephaly (2 standard deviations below the mean) and early FGR was diagnosed with Williams syndrome. An overview of the clinical characteristics of the prenatal cases is presented in Table 1. The prenatal cohort consisted of 73.3% female fetuses, significantly higher than the expected female-to-male ratio (*p* < 0.05). In the majority of cases (73.3%), microcephaly co-occurred with other findings. We evaluated whether clinical parameters such as fetal sex, the presence of additional sonographic findings, and specific sonographic findings like brain malformations, heart malformations, abnormal fetal growth, and abnormal amniotic fluid measurements correlated with an abnormal CMA result and found no significant associations (Table 2).

**Table 1:** Clinical characteristics of the 87 prenatal cases

Characteristic	Mean ± SD
Maternal age (years)	31.5 ± 5.2
Paternal age (years)	32.7 ± 5.3
Gestational age at amniocentesis (weeks)	31.9 ± 4.2
Additional findings	64 (73.6)
FGR^a^	44 (5.1)
Brain malformations^b^	8 (9.2)
CHM^c^	6 (6.9)
Other malformations^d^	5 (5.7)
Soft signs^e^	11 (12.6)
Oligohydramnios^f^	11 (12.6)
Polyhidramnious^g^	1 (1.1)
Dysmorphic features^h^	3 (3.4)
Short femur^i^	5 (5.7)
Fetal gender	
Male	23 (26.4)
Female	64 (73.6)
CMA positive findings	4 (4.6)

*FGR* fetal growth restriction, *CHM* congenital heart malformations

^a^FGR-estimated fetal weight below the 3rd percentile

^b^Brain malformations including abnormal sulcation, corpus callosum abnormalities, ventriculomegaly, polymicrogyria

^c^CHM-ventricular septal defect, aberrant right subclavian artery

^d^Other malformations including kidney, genitalia, clubfoot, lung malformations

^e^Soft markers for Down syndrome were echogenic focus at the left ventricle of the fetal heart, echogenic bowel, single umbilical artery

^f^Amniotic fluid index below the 3rd percentile for gestational age

^g^Amniotic fluid index above the 97th percentile for gestational age

^h^Dysmorphic features including abnormal cranium shape, malformed ears

^h^Femur length below the 3rd percentile

**Table 2:** CMA Yield by clinical parameters in 87 prenatal cases

Parameter	CMA			*P*-value
	Normal *N* (%)	Abnormal *N* (%)	Total	
Female fetus	61 (95.31)	3 (4.69)	64	0.95
Male fetus	22 (95.65)	1 (4.35)	23	
Isolated	21 (91.30)	2 (8.70)	23	0.27
Non-isolated	62 (96.88)	2 (3.12)	64	
No-FGR	41 (95.35)	2 (4.65)	43	0.98
FGR^a^	42 (95.45)	2 (4.55)	44	
No-brain mlaformations	76 (96.2)	3 (3.8)	79	0.26
Brain malformations^b^	7 (87.50)	1 (12.5)	8	
No CHM	77 (95.06)	4 (4.94)	81	0.58
CHM^c^	6 (100.00)	0	6	
No dysmorphic features	80 (95.24)	4 (4.82)	84	0.7
Dysmorphic features^d^	3 (100.00)	0	3	
No-short femur	78 (95.12)	4 (4.88)	82	
Short femur^e^	5 (100.00)	0	5	0.61
No-soft signs	72 (94.74)	4 (5.26)	76	
Soft signs^f^	11 (100)	0	11	0.44
No-olygohydramnious	72 (94.74)	4 (5.26)	76	
Olygohydramnious^g^	11 (100%)	0	158	0.44
No-polyhydramnious	82 (95.35)	4 (4.60)	86	
Polyhydramnious^h^	1 (100.00%)	0	1	1.0

^a^FGR-fetal growth restriction-estimated fetal weight below the 3ed percentile

^b^Brain malformations including abnormal sulcation, corpus callosum abnormalities, ventriculomegaly, polymicrogyria,

^c^CHM—congenital heart malformations: Ventricular septal defect, Aberrant right subclavian artery

^d^Dysmorphic features including abnormal cranium shape, Malformed ears

^e^Femur length below the 3rd percentile

^f^Soft markers for Down syndrome were echogenic focus at the left ventricle of the fetal heart, echogenic bowel, single umbilical artery

^g^Amniotic fluid index below the 3rd percentile for gestational age

^h^Amniotic fluid index above the 97th percentile for gestational age

The CMA yield was notably higher in the cohort of prenatally-detected microcephaly cases when compared to the background risk in uncomplicated pregnancies (*p* = 0.013).

### CMA for postnatally detected microcephaly

Out of the 742 cases included in the postnatally detected microcephaly cohort, 111 (15.07%) exhibited a pathogenic result, of which only one was detectable by karyotype.

Table 3 provides an overview of the clinical characteristics of the postnatal cases. Notably, in most cases (97.3%), microcephaly was accompanied by other findings.

**Table 3:** Clinical characteristics of the cohort of 742 postnatal cases

Characteristic	Mean ± SD
Age (years)	7.59 ± 10.45
	*N (% of 743)*
Isolated cases	20 (2.7)
Intellectual disability/developmental delay	546 (73.6)
Autism	50 (6.7)
Hypotonia	75 (10.1)
Epilepsy	98 (13.2)
ADD/ADHD^a^	23 (3.1)
Learning disability	3 ( 0.4)
Other neurologic phenotypes^b^	45 (6.1)
Dysmorphic features	219 ( 29.5)
Growth abnormality^c^	204 (27.5)
Brain malformations^d^	67 (9.0)
CHM^e^	71 (9.6)
Other malformations^f^	90 (12.2)
Hearing loss	23 (3.1)
Abnormal findings during the pregnancy	
FGR^g^ during the pregnancy	17 (2.3)
Gender	
Male	393 (53)
Female	349 (47)
CMA positive finding	111 (15.0)

^a^ADD/ADHD-attention deficit disorder/attention deficit hyperactivity disorser

^b^Other neurologic phenotypes: movment disorders, spasticity, spastic paraplegia, muscle weakness, dystonia, ataxia, areflexia

^c^Growth abnormality: failure to thrive, short stature

^d^Brain malformations: corpus callosum malformations, migration disorders, leukomalacia, cerebellar abnormalities/ vermal disorders, white matter abnormalities, ventriculomegaly, atrophy

^e^CHM—congenital heart malformations: Ventricular septal defect, Atrial septal defect, Aortic coarctation, Patent ductus arteriosus, Right aortic arch, AV canal, Tetralogy of fallot, Pulmonic stenosis, Aortic stenosis

^f^Other malformations: All other malformations such as kidney malformations, palate malformations, limb malformations, diaphragmatic hernia, clubfoot, Choanal atresia

^g^FGR-fetal growth restriction-estimated fetal weight below the 3rd percentile during the pregnancy

We assessed whether clinical parameters such as sex, the presence of neurologic clinical phenotypes, brain malformations, other malformations, and other phenotypes were correlated with an abnormal CMA (Table 4**)**.

**Table 4:** CMA yield by clinical parameters in 742 postnatal cases

Parameter	CMA			*P*-value
	Normal *N* (%)	Abnormal *N* (%)	Total	
Female	282 (83.2)	57 (16.8)	339	0.25
Male	339 (86.3)	54 (13.7)	393	
Isolated	19 (95)	1 (5.0)	20	0.21
Non-isolated	612 (84.8)	110 (15.2)	722	
No-intellectual disability	161 (82.1)	35 (17.9)	196	0.18
Intellectual Dissability	470 (86.1)	76 (13.8)	546	
No- autism	590 (85.3)	102 (14.7)	692	0.76
Autism	41 (83.7)	8 (16.3)	49	
No hypotonia	573(85.9)	94 (14.1)	667	0.05 *
Hypotonia	58 (77.3)	17 (22.7)	75	
No epilepsy	554 (86.0)	90 (14.0)	644	0.05 *
Epilepsy	77 (78.6)	21 (21.4)	98	
No-learning disability	630 (85.3)	109 (14.7)	739	0.01*
Learning dissability	1 (33.3)	2 (66.7)	3	
No-dysmorphysm	457 (85.9)	75 (14.1)	532	0.03*
Dysmorphysm	174 (79.4)	45 (20.6)	219	
No-brain malformations	570 (84.3)	105 (15.8)	666	0.15
Brain Malformations	61 (91.0)	6 (9.0(	67	
No-CHM^a^	580 (86.4)	91 (13.6)	671	0.00*
CHM	51 (71.8)	20 (28.2)	71	
No-FGR^b^ during pregnancy	620 (85.5)	105 (14.5)	725	0.02*
FGR during pregnancy	11 (64.7)	6 (35.3)	17	

^a^*CHM* congenital heart malformations

^b^FGR-fetal growth restriction-estimated fetal weight below the 3rd percentile

^*^Statistically significant

A significantly higher detection rate for CMA was detected in cases with dysmorphism, hypotonia, epilepsy, congenital heart malformations (CHM), learning disabilities (LD), and a history of Fetal growth restriction (FGR).

No specific recurrent copy number variations (CNVs) were observed.

The rate of variants of unknown significance was 3.9%.

Table 5 presents characteristics of all detected CNVs.

**Table 5:** Characteristics of 116 detected CNVs in the total 829 cases

	Pathogenic and LP CNVs out of total cohort	Karyotype detectable	Known recurrent syndrome	In Unique variants -involvement of a known Monogenic (Autosomal Dominant) genes	Additional CNVs	Deletion
Prenatal	4/87 4.6%	1/4 25%	3/4 75%	0/4 0%	1/4 25%	2/4 50%
Postnatal	112/743 15.%	23/112 20.5%	64/112 57%	27/112 24%	21/112 18.7%	74/112 67%
Total	116/830 14.0%	24.1/116 20.7%	67/116 57.7%	27/116 23.2%	22/116 19%	76/116 66.4%

The list of CNVs and clinical characteristics of the positive for the entire cohort is provided in supplementary Table 1.

## Discussion

In the present study, among the 829 CMA tests performed on microcephaly cases, 13.9% yielded pathogenic/ LP results, with only 18% potentially detectable by karyotype analysis. In the prenatal cohort of 87 cases, 4.6% showed pathogenic results, with only one being detectable by karyotype. It is noteworthy that most of the study population underwent initial screening for trisomy using NT and biochemical markers in the first and second trimesters. Only one case in our cohort had abnormal biochemical screening results but a normal CMA analysis, what might explain the relatively low CMA yield in our study.

To the best of our knowledge, there are limited data regarding the yield of CMA performed due to sonographic diagnosis of microcephaly. Sheffer et al. [12] examined the yield of CMA in pregnancies with abnormal ultrasounds and stratified them according to the specific abnormal sonographic findings. A pathologic result was detected in 1/32 (3.1%) of isolated cases of microcephaly and in 1/5 (20%) of cases with additional sonographic abnormality, yielding a total of 2/37 (5.4%) pathogenic CMA among all patients with fetal microcephaly. Liu et al. [20] assessed the yield of genetic testing performed in 157 fetuses with HC <–2 SD. The overall diagnostic yield of CMA was 13% with a significantly higher yield in non-isolated cases. In another study by Wang et al. [21] the diagnostic yield of CMA among 187 fetuses with prenatal microcephaly was 3.74%.

The CMA yield in this study was notably higher in the cohort of prenatally-detected microcephaly cases when compared to the background risk in uncomplicated pregnancies suggesting that CMA testing should be done in cases with prenatally-detected microcephaly.

There are a few reports regarding genetic testing for postnatal cohorts of microcephaly. Bonnsawat et al. [8] assessed 62 patients with primary and secondary microcephaly using high resolution CMA analysis, exome sequencing and functional studies and detected a causative variant in 48%. The rate of CNVs in this cohort was 9.7% (6 cases). It is important to mention, though, that 90% of the study population had co-morbidity of developmental delay and 85% of the patients had intellectual disability.

In a cohort of 680 children with microcephaly [7], the putative etiology was ascertained in 59%. Genetic causes were detected in 14.8% (chromosomal microscopic and submicroscopic aberrations in 6.8%, monogenic conditions in 8%) and a putative genetic cause was suspected in 13% based on the phenotype or family history.

Shaheen et al. [10] assessed 150 cases of congenital microcephaly with 56 mendelian forms using exome sequencing. They reported little overlap with the genetic causes of postnatal microcephaly.

It is expected that the yield of prenatally-detected microcephaly will be much lower than for postnatal cases due to a number of variables, including the accuracy of the diagnosis, which is suboptimal in prenatal cases and selection bias of cases being evaluated.

In this study, the yield for CMA was 4.6% for the prenatal cohort, and 15% for the postnatal cohort. Interestingly, in the prenatal cohort 73.6% of cases were non-isolated while in the postnatal cohort 97% were non- isolated. However, there is a difference between prenatal and postnatal non-isolated cases. In the prenatal setting, many additional findings are sonographic anomalies that are only present during the pregnancy whereas many additional findings reported in the postnatal setting such as neurocognitive abnormalities are not detectable prenatally. The risk for abnormal CMA was higher for postnatally detected non-isolated microcephaly cases, however, this observation did not reach a statistical significance. In the prenatal cohort the rate of abnormal CMA was higher in isolated cases however the numbers were too small to draw any conclusions from this observation.

Another noteworthy finding is the predominance of females among fetuses with microcephaly. It is important to note that fetal gender and head circumference biometry charts typically do not differentiate between male and female fetuses. However, postnatally and at birth, there are variations in normal OFC values between males and females. A recent study conducted in Singapore even proposed the use of gender-specific charts for fetal head circumference to enhance the accuracy of microcephaly detection [22]. The higher proportion of females in our microcephalic cohort implies that the implementation of gender-customized charts may prove beneficial. A similar male-to-female ratio was observed in a study on prenatally-detected macrocephaly, where 86.4% of fetuses diagnosed with isolated macrocephaly were male [23]. Another cohort that assessed the yield of CMA among cases with abnormal prenatally-detected HC also reported the same disproportion with 87% of microcephaly cases detected in female fetuses and 86% of macrocephaly cases reported in male fetuses [24].

A few studies found that female fetuses present smaller HC, starting from the second trimester of pregnancy [25–28]. In a recent study, gender-customized curves based on a cohort of 11,404 fetal measurements showed that the male HC curve was significantly higher than the female curve for all gestational weeks [28]. Yet, most centers do not use gender-specific charts for HC during gestation. Therefore, it is possible that over-diagnosis of microcephaly among females and under-diagnosis among males occurs, and the opposite for macrocephaly. This may lead to inaccurate diagnoses and may explain the very large differences in genetic evaluation yields for microcephaly and macrocephaly between fetuses and children.

Our cohort included a larger number of postnatal patients compared to prenatal cases.

This discrepancy can be attributed to several factors. First, obtaining prenatal samples involves invasive procedures, whereas postnatal testing is performed in blood samples. Also, microcephaly is usually diagnosed late in the pregnancy and many couples choose not to proceed with genetic testing while the motivation for postnatal genetic evaluation is usually high especially when significant additional findings are present. Also, only primary microcephaly is included in prenatally-detected cases.

This study has limitations due to its retrospective nature, impacting data collection. Furthermore, both cohorts were small, making it impossible to draw definitive conclusions about the exact yield of CMA testing for microcephaly for different clinical scenarios.

Despite these limitations, we still advocate for the use of CMA testing in cases of prenatally-detected abnormal fetal head parameters. In addition, we strongly recommend using gender-specific growth charts. thisMoreover, in cases where CMA testing yields normal results, but growth parameters deviate significantly from the normal range, we suggest considering additional genetic tests based on next-generation sequencing technologies (gene panels, exome sequencing, and whole genome sequencing), to search for syndromes caused by single gene disorders.

### Supplementary Information

Below is the link to the electronic supplementary material.

Supplementary file1 (DOCX 60 KB)
