# Supplementary material for: Chromosomal microarray testing yield in 829 cases of microcephaly: a clinical characteristics-based analysis for prenatal and postnatal cases
Source: Arch Gynecol Obstet. 2024 Mar 18;310(3):1547–54. doi: 10.1007/s00404-024-07388-3 (PMC11366728; doi:10.1007/s00404-024-07388-3)
Supplement: Supplementary file 1 — Supplementary file1 (DOCX 60 KB) [file 404_2024_7388_MOESM1_ESM.docx]

Supplementary table 1a: Cases with an abnormal CMA result -cohort of prenatal cases

| **Case** | **Classification** | **Additional ultrasound**  **findings** | **Gain/loss** | **Chromosomal cytoband** | **Genomic location**  **(GRCH37/hg19)** | **Size (Mb)** | **Karyotype detectable** | **Recurrent CNV/ syndrome** | **Involvement of a Known gene** | **Secondary CNV** |
| --- | --- | --- | --- | --- | --- | --- | --- | --- | --- | --- |
| 1 | P | No | Gain | 17q12 | chr17:34,815,551-36,185,351 | 1.3 | No | Yes | HNF1B | No |
| 2 | P | Ventriculomegaly, severe early FGR | Gain | Triploidy | NR | NR | Yes | Yes | NR | No |
| 3 | P | Early FGR (15 W) | Loss | 7q11.23 | chr7:72,722,981-74,138,603 | 1.4 | No | Williams-Beuren syndrome | ELN1 | No |
| 4 | P | No | Loss | 2q33.1-q34 | chr2:202,638,054-209,125,186 | 6.5 | No | No | No | GAIN 15q13.1 (1.7MB)  chr15:28,924,405-30,657,952 |

Supplementary table 1b: Cases with an abnormal CMA result -cohort of postnatal cases

| **Case** | **Classification** | **Additional findings** | **Gain/loss** | **Chromosomal cytoband** | **Genomic location**  **(GRCH37/hg19)** | **Size (Mb)** | **Karyotype detectable** | **Known syndrome** | **Involvement of a Known gene** | **Secondary CNV** |
| --- | --- | --- | --- | --- | --- | --- | --- | --- | --- | --- |
| 1 | P | DD, palate insufficiency | Loss | 22q11.21 | chr22:18,874,331-21,722,994 | 2.84 | No | 22q11 proximal deletion syndrome/Velocardiofacial syndrome | TBX1 | No |
| 2 | P | VSD, aniridia | Loss | 4q32.2 | NA | 15.3 | Yes | No |  | Yes  Loss 11p14.1 (2.8 Mb)  Loss 5p13.1  (1.36Mb) |
| 3 | P | ID | Gain | 10q26.2-26.3 | chr10:127,596,011-135,534,747 | 7.9 | No | No | No | No |
| 4 | P | ID, convulsions, dysmorphism | Loss | 4p16.3 | chr4:0-3,809,107 | 3.8 | No | Wolf-Hirschhorn syndrome | NSD2 | No |
| 5 | P | ID dysmorphism hypospadias hypotonia FTT | Loss | 4p | chr4:0-3,800,366 | 3.8 | No | Wolf-Hirschhorn syndrome | NSD2 | No |
| 6 | P | FGR during pregnancy, ID, VSD, hypotonia, dysmorphism, | Gain | Trisomy 21 | chr21:14,613,203-48,129,895 | 33.5 Mb | Yes | Down syndrome | No | No |
| 7 | LP | No | Loss | 7q31.1 | chr7:107,833,066-111,609,725 | 3.7 | No | No | No | No |
| 8 | P | ID, dysmorphism. short stature | Loss | 3q24 | chr3:148,335,613-159,769,643 | 11.4 | Yes | No | No | No |
| 9 | P | Mild ID, FTT, epilepsy, Hepatic Disease | Loss | 20p12.3-p12.1 | chr20:7,373,327-12,989,212 | 5.6 | No | No | JAG1  [Alagille syndrome](https://www.omim.org/entry/118450) | No |
| 10 | P | No | Gain | 16p11.2 | chr16:29,649,915-30,281,111 | 1.12 | No | 16p11.2 proximal duplication syndrome | TBX6 | No |
| 11 | LP | FTT, dimorphism | Loss | 8p23.1 | chr8:8,094,406-9,440,477 | 2.5 | No | 8p23.1 deletion syndrome |  | Loss 8p23.1 (1.3 Mb)  (chr8:12,483,094-15,054,719) |
| 12 | LP | ID | Loss | 16p13.11 | chr16:15,392,742-16,363,239 | 0.9 | No | 16p13.11 proximal deletion syndrome | MYH11 | Gain13q14.12  (2.8 Mb)  (chr13:45,686,940-48,525,200) |
| 13 | LP | ID, ataxia | Loss | 18q23 | chr18:76,436,383-78,077,248 | 1.6 | No | No | No | Gain 18q23 (0.5 Mb)  chr18:74,246,381-74,766,952 |
| 14 | P | ID, ASD, normal karyotype on amniotic fluid | Loss | 10q26 | chr10:125,281,819-135,534,747 | 10 | No | No | EBF3 | No |
| 15 | P | Diaphragmatic hernia, VSD, dysmorphism, unilateral club foot | Loss | 1q41-q42.12 | chr1:216,436,033-225,626,072 | 9.1 | No | Skraban-Deardorff syndrome | WDR26 | No |
| 16 | P | Autism, ADHD | Gain | 7q11.23 | chr7:72,722,981-74,138,603 | 1.4 | No | 7q11.23 duplication syndrome | ELN1 | No |
| 17 | LP | ID, convulsions, encephalopathy | homozygous loss | 16p13.3 | chr16:6,418,301-6,852,490 | 0.4 | No | No | RBFOX1 | No |
| 18 | P | Hypotonia, SGA | Loss | 15q1.2-q13.1 | chr15:22,299,434-28,544,359 | 4.9 | No | Prader-Willi Syndrome | UBE3A | No |
| 19 | P | ID | Loss | 22q11.21 | chr22:18,844,632-21,232,210 | 2.4 | No | 22q11 proximal deletion syndrome/Velocardiofacial syndrome | TBX1 | No |
| 20 | P | ID, ADHD | Gain | 16p11.2 | chr16:29,649,915-30,281,111 | 0.63 | No | 16p11.2 proximal duplication syndrome | TBX6 | No |
| 21 | P | progressive hypotonia, dysmorphism, left CDH | Loss | 5p15 | chr5:0-23,214,968 | 23 | Yes | [Cri-du-chat syndrome](https://medlineplus.gov/genetics/condition/cri-du-chat-syndrome/) | No | No |
| 22 | P | ID, FTT,, dysmorphism | Loss | 1q21.1-q21.2 | chr1:146,501,348-147,826,789 | 1.3 | No | 1q21 distal deletion syndrome | GJA5,GJA8 | No |
| 23 | P | FTT, PS, anterior displacement of anus, hypotonia, developmental delay | Loss | 7q11.23 | chr7:72,722,981-74,138,603 | 1.4 | No | Williams-Beuren syndrome | ELN1 | No |
| 24 | P | FTT, right aortic arch, bicuspid AV, moderate ASD | Loss | 22q.11 | chr22:18,844,632-21,703,145 | 2.8 | No | 22q11 proximal deletion syndrome/Velocardiofacial syndrome | TBX1 | No |
| 25 | P | Peri membranous VSD, ASD, FTT | Loss | 22q.11.21 | chr22:18,844,632-21,536,104 | 2.7 | No | 22q11 proximal deletion syndrome/Velocardiofacial syndrome | TBX1 | No |
| 26 | P | short stature | Gain | 22q.11.21 | chr22:18,844,632-21,461,685 | 2.6 | No | 22q11 proximal duplication syndrome | TBX1 | No |
| 27 | P | ID, dysmorphism, friendly | Loss | 7q11.23 | chr7:72,722,981-74,138,603 | 1.4 | No | Williams-Beuren syndrome | ELN1 |  |
| 28 | P  LP | FTT, ID, seizures | Gain | 22q11.2 | chr22:18,844,635-21,026,935 | 2.1 | No | 22q11 proximal duplication syndrome |  | Del 2q23.1  0.3 Mb  chr2:148,752,586-149,089,645 |
| 29 | P | ID | Loss | del 18q23 | chr18:74,095,640-78,077,248 | 4 | No | No | No | dup 11q24.3-q25  (5.9 Mb)  chr11:128,978,217-134,839,416 |
| 30 | LP | Cerebellar hypoplasia, agenesis of CC, dysmorphism, syndactyly, craniosynostosis albinism | Loss | Xq12 | chrX:67,437,927-68,263,771 | 0.8 | No | no | ENFB1 | No |
| 31 | P | Microcephaly, capillary hemangioma, PS, hypotonia | Gain | XXY | NR | NR | Yes | Klinefelter syndrome | NO | NO |
| 32 | P | Severe ID, hypotonia, dysmorphism | Loss  Gain | 2q37.3 | chr2:238,517,580-243,199,373 | 4.68 | No | No | HEDAC4 | Dup 10p15.3 (6.6 Mb)  chr10:1-6,673,298 |
| 33 | LP | ID, dysmorphism | Loss | 2q36.1 | chr2:223,125,530-223,745,152 | 0.62 | No | no | PAX3 | No |
| 34 | P | TOF, ID, seizures, ptosis, dysmorphism | Gain | 16p13.3 | chr16:2,638,684-4,032,032 | 1.4 | No | 16p13.3 microduplication syndrome | CREBBP |  |
| 35 | LP | ID, seizures, FGR | Gain | 16p13.11 | chr16:15,129,955-16,363,239 | 1.2 | No | 16p13.11 microduplication syndrome | MYH11 | Dup 20q11.21(1.1 Mb)  (chr20:29,507,776-30,619,234  ) |
| 36 | P | ID, FGR, Dysmorphism, Syndactyly | Loss | 1q21 | chr1:146,501,348-147,826,789 | 1.3 | No | 1q21 distal deletion syndrome | GJA5,GJA8 | No |
| 37 | P | FGR, syndactyly, dysmorphism | Loss | 1q21.1 | chr1:146,501,348-147,826,789 | 1.3 | No | 1q21 distal deletion syndrome | GJA5,GJA8 | No |
| 38 | P | ID, seizures, dysmorphism, abnormal brain IDI: colpocephaly, syringomyelia C4 -C5 | Loss | 6q25.3-q27 | chr6:160,731,051-171,115,067 | 10.3 | Yes | No | DLL1 | No |
| 39 | P | ID, seizures | Loss | 1q21.1 | chr1:146,501,348-147,826,789 | 1.3 | No | 1q21 Distal deletion syndrome | GJA5,GJA8 | No |
| 40 | P | Tricaspid atresia, FTT, hypotonia, pectus carinatum | Mosaic gain | 46,XY/47,XXY mosaicism | chrX:1-155,270,560 | 155.2 | Yes | Mosaic kleinfelter syndrome | NR | No |
| 41 | P | ID, short stature | Gain | 2p21 | chr2:91,812,834-105,491,528 | 13.7 | Yes | No | No | No |
| 42 | P | ID, mild dysmorphism | Gain | 16p11.2 | chr16:29,503,993-30,281,111 | 0.77 | No | 16p11.2 proximal duplication syndrome | TBX6 | No |
| 43 | P | ID, hypotonia, dysmorphism | Loss | 15q11.2 | chr15:23,656,946-28,544,359 | 4.9 | No | Prader-Willi Syndrome | UBE3A | Dup 15q13.1 (3.6 Mb)  (chr15:28,924,405-32,620,127 |
| 44 | P | ID, short stature, dysmorphism, bilateral club feet | Gain | 17p11.2 | chr17:16,709,465-20,229,310 | 3.5 | No | Potocki-Lupski syndrome | RAI1 | No |
| 45 | LP | ID | Loss | 16p12.2 | chr16:21,789,785-22,494,295 | 0.7 | No | 16p12.2 distal deletion syndrome | CDR2 | No |
| 46 | P | ID | LOH | UPD whole chromosome 15  15q11.2q26.3 | chr15:20,161,372-102,531,392 | 82 | No | Prader-Willi/ AngelmanSyndrome | No | No |
| 47 | P | ID severe, hypotonia | Loss | 6q11.1-q14.3 | chr6:62,149,721-87,522,387 | 25.3 | Yes | No | PHIP | No |
| 48 | LP | Seizures (rolandic epilepsy) | Loss | 16p12.2 | chr16:21,894,231-22,422,637 | 0.6 | No | 16p11.2 distal deletion syndrome | CDR2 | No |
| 49 | LP | ID, dysmorphism, macrodontia | Loss | 10p12.1 | chr10:25,848,265-29,343,641 | 3.5 | No | No | WAC | No |
| 50 | P | ID, dysmorphism, mental illness | Loss | 16p13.1 | chr16:15,454,755-16,363,239 | 0.9 | No | 16p13.11 microdeletion syndrome | MYH11 | No |
| 51 | P | ID, hypotonia, dysmorphism, hydrocephalus, seizures, abnormal ears | Loss | 1p36.33-p36.2 | chr1:752,566-2,558,903 | 1.8 | No | No | No | No |
| 52 | P | ID, partial agenesis of CC, ataxia, mild dysmorphism | Loss | 1q43-44 | chr1:243,556,929-244,794,846 | 1.2 | No | No | AKT3 | No |
| 53 | LP | DD, FTT, FGR, retrognathia, dysmorphism, hypotonia | Loss | 7q11.22-q11.23 | chr7:70,196,741-72,305,671 | 2.1 | No | No | AUTS2 | No |
| 54 | P | DD, abnormal eye contact | Loss | 18q21.31-q23 | chr18:54,406,909-78,077,248 | 23.7 | Yes | No | No | No |
| 55 | P | ID, ADHD, hearing impairment | Gain | 16p11.2 | chr16:29,503,993-30,206,886 | 0.7 | No | 16p11.2 proximal duplication syndrome | TBX6 | No |
| 56 | LP | ID, dysmorphism, hypogonadism | Loss | 3q26.33 | chr3:181,414,820-181,595,814 | 0.18 | No | No | SOX2 | No |
| 57 | P | ID, seizures, , ataxia | Loss | 15q11.2 | chr15:23,656,946-28,544,359 | 4.8 | No | Angelman syndrome | UBE3A | No |
| 58 | P | ID, port- wine stain | Loss | 20q13.3 | chr20:61,809,828-63,025,520 | 1.2 | No | No | KCNQ2 | No |
| 59 | P | ID, seizures | Loss | 1q21.1-q21.2 | chr1:146,456,447-148,003,653 | 1.5 | No | 1q21 Distal deletion syndrome | GJA5,GJA8 | No |
| 60 | P | ID, seizure, obesity | Loss | 16p11.2 | chr16:29,503,993-30,289,846 | 0.7 | No | 16p11.2 proximal deletion syndrome | TBX6 | No |
| 61 | P | DD, dysmorphism, webbed neck, congenital heart malformation: AV canal, PDA- severe heart failure | Gain | 11q23.3-q25 | chr11:116,676,862-135,006,516 | 18.3 | Yes | No | No | Gain 22q11.1-q11.21  (3.2 Mb)  (chr22:17,012,935-20,312,668) |
| 62 | P | Dysmorphism, VSD | Gain | 17q12 | chr17:34,810,470-36,185,351 | 1.4 | No | 17q12 duplication syndrome | HNF1B | No |
| 63 | P | FTT (-4SD), short stature | Gain | XXY | chrX:1-155,270,560 | 155 | Yes | kleinfelter syndrome | No | No |
| 64 | P | DD, dysmorphism, congenital heart malformation: ASD, hypotonia | Gain | 3p26.1 | chr3:4,330,032-28,430,692 | 24 | Yes | No | No | No |
| 65 | LP | DD, hearing loss, immune deficiency | Loss | 4q35.1 | chr4:185,399,431-191,154,276 | 5.7 | No | No | No | No |
| 66 | P | DD, FTT, hypotonia, dysmorphism, hearing impairment | Loss | 5q15 | chr5:92,929,099-120,190,815 | 27.2 | Yes | No | NR2F1, APC | Loss 7p21.3 (0.9 Mb)  (chr7:7,948,926-8,852,949) |
| 67 | LP | Microphthalmia, retinal disorder, eye coloboma (right), eye nystagmus (left), cognitive impairment, hypotonia, facial cleft | Gain | 3q13.2 | chr3:113,485,257-114,009,675 | 0.524 | No | No | ATP6V1A | No |
| 68 | P | ID | Gain | 17q11.2 | chr17:28,983,942-30,408,079 | 1.42 | No | NF1 microduplication syndrome | NF1 | No |
| 69 | P | ID, seizures, abnormal brain IDI | Loss | 6q16.1-q21 | chr6:99,185,586-108,118,361 | 8.9 | No | No | No | No |
| 70 | LP | ID, dystonia | Loss | 16q24.3 | chr16:89,813,235-90,354,753 | 0.541 | No | No | TUBB3 | No |
| 71 | P | FGR, rocker feet | Loss | 4p16.3 | chr4:0-13,496,535 | 13 | Yes | No | No | Gain 4p15.33 (35.6Mb) (chr4:13,496,535-49,109,992) |
| 72 | LP | ID, FTT, VSD, FGR | Loss | 21q21.2 | chr21:25,733,979-31,304,563 | 5.5 | No | No | No | Loss 21.21 (3.4 Mb) (chr21:19,914,953-23,363,182) |
| 73 | LP | ID, FTT, hypertonus | Loss | 7q11.22 | chr7:69,919,065-70,253,486 | 0.334 | No | No | AUTS2 | No |
| 74 | P | ID, hearing impairment right, cryptorchidism unilateral, inguinal hernia unilateral, dysmorphism | Gain | 14q32.12-q32.33 | chr14:94,209,931-107,349,540 | 13.1 | Yes | No | DYNC1H1 | Loss 11q24.3-q25  (5 Mb)  (chr11:129,980,872-135,006,516) |
| 75 | P | DD, palate insufficiency | Loss | 22q11.21 | Chr22: 18,874,331-21,722,994 | 2.84 | No | 22q11 proximal deletion syndrome/Velocardiofacial syndrome | TBX1 | No |
| 76 | LP | Autism, Facial dysmorphism | Loss | 12q24.23q24.31 | Chr12: 120,666,064-121,761,489 | 1.1 | No | No | HNF1A | No |
| 77 | P | Autism, Facial dysmorphism, Short stature, Mental retardation, | Gain | 21q11.2q22.11 | Chr21:15,342,577-34,169,207 | 18.82 | Yes | No | No | No |
| 78 | P | Autism, Developmental delay | Loss | 22q11.21 | Chr22: 21,804,596-22,962,962 | 1.1 | No | 22q11.2 distal deletion syndrome | MAPK1 | Gain 22q11.21  0.7 Mb  (chr22: 21,029,656-21,800,471) |
| 79 | P | Facial dysmorphism, Cleft palate, Abnormal heart morphology | Gain | 22q11.21 | Chr22: 16,888,899-20,312,661 | 3.42 | No | 22q11 proximal duplication syndrome | TBX1 | Gain 18.24 Mb  11q23.3  Chr11: 116,697,903-134,937,416) |
| 80 | P | Developmental delay, Short stature | Gain | 17q12 | Chr17: 34,822,465-36,243,365 | 1.4 | No | 17q12 duplication syndrome | HNF1B | No |
| 81 | P | Developmental delay, Failure to thrive | Loss | 7q11.23 | Chr7:72,621,345-74,207,565 | 1.6 | No | Williams-Beuren syndrome | ELN1 | Loss 7q11.23  (0.5 Mb)  (chr7:74,530,395-75,045,079) |
| 82 | P | Developmental delay, Failure to thrive | Loss | 15q11.2-q13.1 | Chr15: 22,770,421-29,062,448 | 6.29 | No | Prader-Willi Syndrome | UBE3A | No |
| 83 | P | Developmental delay | LOH | UPD whole chromosome 15  15q11.2q26.3 | Chr15: 22,817,870-102,397,317 | 79 | No | Prader-Willi Syndrome | UBE3A | No |
| 84 | P | Developmental delay, Short stature | Gain | 5q35.2q35.3 | Chr5: 175,469,493-177,416,986 | 1.95 | No | No | NSD1 | No |
| 85 | P | Developmental delay, Facial dysmorphism | Loss | 22q11.22 | Chr22: 22,997,928-23,649,562 | 0.652 | No | 22q11.2 distal deletion syndrome | No | No |
| 86 | P | Developmental delay, Short stature, Seizures, Short thumb, Skeletal dysplasia | Loss | 18p11.32p11.21 | Chr18: 136,227-15,170,636 | 15 | Yes | No | TGIF1, SMCHD1 | No |
| 87 | P | Developmental delay, Facial dysmorphism | Loss | 13q33.1q34 | Chr13: 104,753,418-115,107,733 | 10.35 | Yes | No | COL4A1, CHAMP1 | No |
| 88 | P | Developmental delay, Facial dysmorphism, total anomalous pulmonary venous return | Gain | 16p11.2 | Chr16: 28,486,805-30,177,240 | 1.69 | No | 16p11.2 microduplication syndrome (proximal+partial distal) | SH2B1, TBX6 | No |
| 89 | P | Specific learning disability, Facial dysmorphism, Micrognathia | Loss | 7q11.23 | Chr7: 72,723,370-74,154,527 | 1.43 | No | Williams-Beuren syndrome | ELN1 | No |
| 90 | P | Developmental delay, Facial dysmorphism, Failure to thrive | Loss | 1q21.1q21.2 | c146,02hr1: 3,922-147,830,830 | 1.8 | No | 1q21.1 distal microdeletion syndrome | GJA5, GJA8 | No |
| 91 | LP | Mental retardation, Failure to thrive, Club foot | High copy Gain | 7q36.1 | Chr7: 151,664,642-152,578,85 | 0.914 | No | No | KMT2C | No |
| 92 | P | Mental retardation, Developmental delay, Seizures, Hyperpigmentation of the skin | Loss | 7q11.23q21.11 | Chr7: 74,859,637-78,525,810 | 3.7 | No | 7q11.23 microdeletion syndrome | No | No |
| 93 | P | Developmental delay, Facial dysmorphism, Ataxia, Shivering | Loss | 10q26.2q26.3 | Chr10: 130,252,400-135,426,386 | 5.2 | No | No | EBF3 | No |
| 94 | LP | Developmental delay | Gain | 13q31.3q32.3 | Chr13: 93,597,328-99,483,579 | 5.88 | No | No | No | No |
| 95 | LP | Right Aortic arch, facial dysmorphism | Loss | 13q31.2 | Chr13: 88,314,224-94,780,140 | 6.46 | No | No | No | No |
| 96 | P | Seizures, specific learning disability | Gain | 16p11.2 | Chr16: 29,632,721-30,176,508 | 0.544 | No | 16p11.2 proximal microdeletion syndrome | TBX6 | No |
| 97 | LP | Seizures | Loss | 20q13.33 | Chr20: 61,817,932-62,336,671 | 0.51 | No | No | KCNQ2 | No |
| 98 | P | Facial dysmorphism, VSD | Gain | 3q25.32q29 | Chr3: 158,951,695-197,851,444 | 38.9 | Yes | No | No | Loss 6q27  (4.1 Mb)  (chr6:166,816,700-170,914,297) |
| 99 | P | Seizures | Loss | 7q36.1 | Chr7: 148,875,768-151,892,497 | 3 | No | No | KMT2C | No |
| 100 | P | FTT, micropenis, Metopic craniosynostosis | Loss | 1q21.1q21.2 | Chr1: 146,096,700-147,885,600 | 1.79 | No | Distal 1q21 microdeletion syndrome | GJA5,GJA8 | No |
| 101 | P | Facial dysmorphism, hypotonia, hypertelorism, double collecting system | Gain | 18q11.2q23 | Chr18: 20,790,944-78,013,728 | 57.2 | Yes | No | No | No |
| 102 | P | FTT, laryngeal WEB | Loss | 22q11.21 | Chr22: 19,024,793-21,464,764) | 2.4 | No | 22q11 proximal deletion syndrome/Velocardiofacial syndrome | TBX1 | No |
| 103 | P | Lissencephaly | Loss | 17p13.3 | Chr17: 1,203,449-2,577,091 | 1.37 | No | Miller-Dieker syndrome | PAFAH1B1 | No |
| 104 | P | Frontal bossing, facial dysmorphism, hypotonia | Gain | 11p15.5p15.4 | Chr11: 230,680-4,806,668 | 4.57 | No | Beckwith-Wiedemann syndrome | IGF2 | No |
| 105 | P | Cardiovascular malformations | Loss | 22q11.21 | Chr22:18,919,477-20,312,661 | 1.39 | No | 22q11.2 partial (A-B) proximal deletion syndrome | TBX1 | No |
| 106 | P | Café-au-lait spots | Gain | 16p11.2 | Chr16: 29,567,296-30,243,606 | 0.676 | No | 16p11.2 proximal duplication syndrome | TBX6 | No |
| 107 | P | Hearing impairment, complete AV canal defect, double outlet right ventricle | Loss | 22q11.21 | Chr22:18,648,855-21,915,207 | 3.26 | No | 22q11 proximal deletion syndrome/Velocardiofacial syndrome | TBX1 | No |
| 108 | P | Coloboma, VSD, tricuspid atresia, skin tags | Loss | 1q21.1 | Chr1: 146,023,922-147,830,830 | 1.8 | No | Distal 1q21 microdeletion syndrome | GJA5,GJA8 | High copy gain 22q11.1q11.21 (2.1 Mb)  (chr22: 16,888,899-19,003,154) cat eye syndrome |
| 109 | LP | Mental retardation, facial dysmorphism, seizures | Loss | Xp22.33 | Chrx: 2,693,466-3,384,484 | 0.691 | No | No | ARSE | Gain Yp11.31p11.2  (3.5 Mb)  Chry: 2,650,424-6,150,513  gain  Yp11.2  (2.1 Mb) (chry:7,399,680-9,526,559) |
| 110 | P | Developmental delay, mental retardation, facial dysmorphism, seizures | Gain | Trisomy21 | Chr21: 15,342,577-48,097,372 | 32 | Yes | Down syndrome | No | No |
| 111 | P | short stature, ADHD | Gain | 17q12 | Chr17: 34,822,465-36,243,365 | 1.4 | No | 17q12 microduplication syndrome | HNF1B | No |

FGR- fetal growth restriction -estimated fetal weight below the 3ed percentile.

DD- developmental delay

VSD- ventricular septal defect

ID- intellectual disability

FTT- failure to thrive

ASD- atrial spectrum defect

ADHD- attention deficit hyperactivity disorder

SGA-small for gestational age

CDH-congenital dislocation of hips

PS- pulmonic stenosis

CC- corpus callosum

TOF- tetralogy of Fallot

PDA – patent ductus arteriosus
